# Reminiscence and Digital Storytelling to Improve the Social and Emotional Well-Being of Older Adults With Alzheimer’s Disease and Related Dementias: Protocol for a Mixed Methods Study Design and a Randomized Controlled Trial

**DOI:** 10.2196/49752

**Published:** 2023-09-07

**Authors:** Ling Xu, Noelle L Fields, Kathryn M Daniel, Daisha J Cipher, Brooke A Troutman

**Affiliations:** 1 School of Social Work University of Texas at Arlington Arlington, TX United States; 2 University of Texas at Arlington Arlington, TX United States; 3 United States Air Force Academy Colorado Springs, CO United States

**Keywords:** Alzheimer’s disease and related dementias, ADRD, digital storytelling, DST, intergenerational reminiscence, older adult, young adult, randomized controlled trial

## Abstract

**Background:**

Increasing attention is being given to the growing concerns about social isolation, loneliness, and compromised emotional well-being experienced by young adults and older individuals affected by Alzheimer disease and related dementias (ADRD). Studies suggest that reminiscence strategies combined with an intergenerational approach may yield significant social and mental health benefits for participants. Experts also recommended the production of a digital life story book as part of reminiscence. Reminiscence is typically implemented by trained professionals (eg, social workers and nurses); however, there has been growing interest in using trained volunteers owing to staffing shortages and the costs associated with reminiscence programs.

**Objective:**

The proposed study will develop and test how reminiscence offered by trained young adult volunteers using a digital storytelling platform may help older adults with ADRD to improve their social and emotional well-being.

**Methods:**

The proposed project will conduct a randomized controlled trial to assess the effects of the intervention. The older and young adult participants will be randomly assigned to the intervention (reminiscence based) or control groups and then be randomly matched within each group. Data will be collected at baseline before the intervention, in the middle of the intervention, at end of the intervention, and at 3 months after the intervention. An explanatory sequential mixed methods design will be used to take advantage of the strengths of both quantitative and qualitative methods. The quantitative data from surveys will be entered into SPSS and analyzed using covariate-adjusted linear mixed models for repeated measures to compare the intervention and control groups over time on the major outcomes of participants. Conventional content analysis of qualitative interviews will be conducted using data analysis software.

**Results:**

The project was modified to a telephone-based intervention owing to the COVID-19 pandemic. Data collection started in 2020 and ended in 2022. In total, 103 dyads were matched at the beginning of the intervention. Of the 103 dyads, 90 (87.4%) dyads completed the midtest survey and 64 (62.1%) dyads completed the whole intervention and the posttest survey. Although we are still cleaning and finalizing data analyses, the preliminary results from both quantitative and qualitative data showed promising results of this intergenerational reminiscence approach that benefits both the older adults who have cognitive impairments and the young adult participants.

**Conclusions:**

Intergenerational reminiscence provided by young adult college student offers promising benefits for both the younger and older generations. Future studies may consider scaling up this pilot into a trackable, replicable model that includes more participants with diverse background (eg, public vs private college students and older adults from other agencies) to test the effectiveness of this intervention for older adults with ADRD.

**Trial Registration:**

ClinicalTrials.gov NCT05984732; https://classic.clinicaltrials.gov/ct2/show/NCT05984732

**International Registered Report Identifier (IRRID):**

DERR1-10.2196/49752

## Introduction

### Background

The projected number of individuals aged ≥65 years living with Alzheimer disease and related dementias (ADRD) is expected to reach 7.1 million by 2025, marking a 22% increase from the current figure of 5.8 million older adults affected [1]. Unless significant advancements in medical research lead to preventive measures, treatments, or cures for ADRD, these statistics are projected to escalate to 13.8 million by 2050 [1]. In addition to memory loss, difficulties in daily functioning, and behavioral symptoms, ADRD profoundly affects the social and emotional well-being of those living with the condition.

Most older adults with ADRD live in the community, and many of these individuals live alone [1,2]. There is growing concern about social isolation and loneliness among people with ADRD, particularly as both have been widely linked to poor physical health outcomes and compromised quality of life and well-being [3]. Studies suggest that people with ADRD are at great risk for social withdrawal, social isolation, and loneliness [4]. Individuals with ADRD also tend to have few close friends, which may negatively affect their quality of life [5]. In addition, ADRD is associated with increased risk of depression; anxiety; and other negative emotional well-being outcomes [6], including decreased resiliency, which may prevent individuals from recovering from life’s challenges [7,8]. Although there are no interventions to prevent or cure ADRD, there is a pressing need to address and understand the social and emotional well-being of people with the disease.

However, people of all ages may experience social isolation and loneliness. The younger generation has various sources that caused their loneliness. It has been reported that Generation Z (aged 18-22 years) is the loneliest generation and claims to be in worse health than older generations [9]. Overall, 7 out of 10 respondents feel alone (73%), shy (72%), or that no one really understands them well (71%) [9]. In the workplace, they also feel abandoned by their coworkers when under pressure at work (42%), alienated by their coworkers (40%), emotionally distant from the people they work with (54%), and disconnected from others at work (55%) [9]. Moreover, young adults were reported to experience heightened levels of loneliness and mental health challenges during the COVID-19 pandemic [10]. Neglecting the issue of social isolation and loneliness can have detrimental effects on both the mental and physical well-being of individuals, as evidenced by studies such as that by Mushtaq et al [11]. Therefore, it is crucial to develop programs or interventions that aim at preventing or addressing loneliness, so that physical and mental health of young and older adults is maintained.

### Reminiscence Approach

Reminiscence has been identified as a potentially effective intervention strategy for alleviating depression [12-14] and in promoting social and emotional well-being for older adults [15]. Reminiscence typically involves a discussion of past activities, events, and experiences aided by the use of familiar items and objects such as photographs and music [16]. Reminiscence work with people with ADRD is viewed as a psychosocial intervention with a credible evidence base [17]. There is also evidence suggesting that individual reminiscence work that involves a life review and subsequent production of a life story book is associated with improvements in the well-being of people with ADRD [18].

### Intergenerational Reminiscence Approach

Studies suggest that reminiscence combined with an intergenerational approach may yield significant social and mental health benefits for young and older adults. Through intergenerational reminiscence, older people may pass on their life experiences and life lessons to younger generations [19,20], and the sharing of autobiographical memories has both social and psychological functions [21]. Previous studies also assert that the use of an intergenerational approach is an effective strategy for promoting positive social and mental health in later life, such as reducing social isolation or loneliness [22]. Establishing intergenerational contact and fostering relationships between older individuals and young adults serve as effective measures to prevent the formation of stereotypes or negative attitudes and to challenge and dispel existing ones [23,24]. In addition, intergenerational programming can enable the younger generation to develop meaningful connections with older adults [23].

Reminiscence is typically implemented by trained professionals (eg, social workers and nurses); however, there has been growing interest in using trained volunteers owing to staffing shortages and the costs associated with reminiscence programs [25]. As part of the efforts to use volunteers rather than professionals, the concept of joining young volunteers and older generations together for intergenerational reminiscence has emerged as a promising area of research. So far, the work of Chung [25] and Gaggioli et al [26] are the only peer-reviewed studies implementing intergenerational reminiscence with young volunteers. Although several benefits were reported for both generations in these 2 studies, the small size of the sample and absence of a control condition limited the study results. The findings underscore the need for more studies related to intergenerational reminiscence that include young adult volunteers, as this approach may potentially offer a more sustainable and cost-effective intervention for implementation in community-based settings. Thus, more studies are needed to better understand and rigorously measure this approach to intergenerational reminiscence interventions, particularly for older adults with ADRD.

### Reminiscence and Digital Storytelling

There are previous studies of reminiscence work that endorse the use of a life story book that are pertinent to the proposed study [18,25]. Recent studies recommend the production of a digital life story book [17] as part of reminiscence. Digital storytelling (DST) is a technology that uses a 2- to 5-minute audio-visual clip combining text, images, music, photographs, voice-over narration, and other audio [27]. Psychological well-being benefits of DST for the general population have been reported in a systematic review [28]. Previous studies also demonstrate the benefits of DST for people with ADRD [29,30]. Studies of intergenerational DST, which combines DST and intergenerational programming, reported positive outcomes including reciprocal relationships, empathy, connection, and confronting agism [31]. However, few studies combined a reminiscence approach and DST or intergenerational DST. Thus, this study is the first one to develop and test how intergenerational reminiscence using a DST platform may help older adults with ADRD and young adults to improve their social and emotional well-being.

### College Student Involvement

The proposed study will include young adult volunteers recruited from University of Texas at Arlington (UTA), a large and diverse institution of higher education. The rationale for including undergraduate and graduate students in the proposed study is that the positive impact of intergenerational learning experiences on students’ academic and personal development is well documented [32]. Previous studies support the value of intergenerational activities in postsecondary education; provide evidence of benefits, reciprocity, and empowerment outcomes [33]; and suggest that these learning experiences may better prepare undergraduate and graduate students for future work with older adults [31]. Studies further suggest that intergenerational learning experiences that involve interactions with people with ADRD also result in positive gains in academic learning and attitude toward older adults among college students [34]. By connecting young adult volunteers with older adults through interactions during the proposed intervention and preintervention training, this proposed study aims to have a positive influence on attitudes toward aging and knowledge about ADRD. Furthermore, with rapidly changing demographics in the United States, there is an urgent need to combat the stereotypes and negative attitudes toward aging and agism and discrimination of older adults that often exists among younger populations [35].

Studies suggest that intergenerational reminiscence is promising for individuals with ADRD; however, these studies are limited by small sample sizes and less rigorous study designs. A recent systematic review of reminiscence therapy provided in the Cochrane Library specifically recommends large-scale, randomized controlled trials that include a life story book component for people with ADRD [17]. Although a few studies have attempted to use young volunteers as part of small-scale intergenerational reminiscence studies [25,26], the use of undergraduate and graduate students is unexamined so far.

### Aims and Research Questions

#### Overview

To fill the abovementioned research gaps, the proposed study aims to develop and evaluate the feasibility and effectiveness of an intergenerational reminiscence approach in improving the social and emotional well-being of older adults with ADRD. The intervention is guided by continuity theory [36], which posits that the reminiscence process guides individuals to adjust and adapt to changes that occur in their lives. Moreover, a continuity theory approach to reminiscence may also facilitate a strong sense of self [37] and has been recently used in other reminiscence interventions [38]. On the basis of literature and the continuity theory, it is hypothesized that the framework of reminiscence combined with an intergenerational approach and DST will help to improve the social and emotional well-being of both generations and help strengthen or build resilience in older adults with ADRD. Specifically, the study addresses 5 goals.

#### Goal 1—To Quantitatively Test the Effectiveness of This Intervention in Improving the Social Well-Being of Older Adults With ADRD and Young Adults

The objectives are to (1) provide a 10-session intervention with reminiscence and DST to older adults by trained young adult volunteers and (2) test whether older or young adults report significant improvement in quality of life or decline in loneliness after the intervention. Hypotheses include the following: (1) older participants in the intervention group will have high levels of quality of life in the middle of and after the intervention compared with their pretest scores and compared with those in the control group and (2) young and older participants in the intervention group will have low levels of loneliness in the middle of and after the intervention compared with their pretest scores and compared with those in the control group.

#### Goal 2—To Quantitatively Test the Effectiveness of This Intervention in Improving the Emotional Well-Being of Older Adults With ADRD

The objectives are to (1) provide a 10-session intervention with reminiscence and DST to older adults by trained young adult volunteers and (2) test whether older adults report significant changes in affect or resilience after the intervention. Hypotheses include the following: (1) older participants in the intervention group will have high positive affect (PA) and low negative affect (NA) in the middle of and after the intervention compared with their pretest scores and compared with those in control group and (2) older participants in the intervention group will have high resilience scores in the middle of and after the intervention compared with their pretest scores and compared with those in control group.

#### Goal 3—To Quantitatively Test Attitudes Toward Aging Among the Young Adult Volunteers

The objectives are to (1) offer opportunities for trained young adult volunteers to engage with older adults in the 10-session intervention and (2) test changes in their attitudes toward aging after the intervention using surveys. Hypotheses include the following: (1) young adult volunteers in the intervention group will have more positive attitudes toward aging after the intervention compared with that before the intervention and (2) young adult volunteers in the intervention group will have more positive attitude toward aging after the intervention compared with those in the control group.

#### Goal 4—To Quantitatively Test Knowledge and Awareness About ADRD Disease Among the Young Adult Volunteers

The objectives are to (1) offer a 3-hour training to the young adult volunteers before the intervention starts, which covers the biological aspects of ADRD and how to communicate and connect interpersonally to people with ADRD, and (2) test changes in their reported knowledge and awareness about ADRD using surveys. Hypotheses include the following: (1) young adult volunteers in the intervention group will report more knowledge and awareness about ADRD disease after the intervention compared with their pretest scores and (2) young adult volunteers in the intervention group will report more knowledge and awareness about ADRD disease compared with those in the control group after the intervention.

#### Goal 5—To Qualitatively Evaluate the Usefulness of This Intervention From the Perspectives of the Dyads

The objectives are to (1) explore and interpret the statistical results obtained in goals 1 and 2 through individual interviews with older adults who report the greatest and least changes in well-being; (2) explore and interpret the statistical results obtained in goals 3 and 4 through individual interviews with young adult volunteers who report the greatest and least changes in attitudes toward aging; and (3) examine dyad participants’ perceptions about the intervention in terms of its quality, benefits, and usefulness.

## Methods

### Study Design

The proposed project will conduct a randomized controlled trial to assess the effects of the intergenerational reminiscence approach with DST on the social and emotional well-being of older adults with ADRD. An explanatory sequential mixed methods design [39] will be used, which first involves collecting quantitative data and analyzing the data to identify subsamples of participants who report the greatest and least changes and then conducting qualitative interview with these subsamples. Half (46/92, 50%) of the dyad sample will receive the reminiscence DST intervention, and the other half (46/92, 50%) of the sample will receive a regular social visit from young adult volunteers without the intervention. Dyads will be randomly assigned to either the intervention or control group after baseline screening of all participants and, subsequently, randomly matched within each group. To further prevent selection bias, statistical techniques such as covariate-adjusted linear mixed models will be used to adjust for covariate imbalance during data analysis.

A total of 92 dyads are required to test the effectiveness of the intervention in this proposed study (refer to power analysis in the *Sample and Recruitment Plan* section). We will recruit participants on a rolling basis until we recruit 92 dyads. At least one-fourth (23/92, 25%) of the total sample of older adults and young adult volunteers will be recruited before they are randomly matched. Upon signing the consent form, participants will be given the chance to engage in the study. They will be provided with guidance throughout the informed consent process and will subsequently undergo a baseline survey in which all measures will be collected blind to group conditions.

The first group will receive the intervention (X), which includes reminiscence and DST components provided by the young adult volunteers, whereas the second group will serve as the sham control group, which will include social visits calls by the young adult volunteers and an unstructured, nondigital recording (eg, scrapbook or journal) of the social visits. To account for the potential influence of attention and social interactions stemming from the intervention, the control activity is intentionally designed to offer comparable social contacts. This ensures that any observed improvements in participants cannot solely be attributed to the intervention itself. The features of the intervention and sham protocols are the same, except that participants in the intervention group will participate in structured reminiscence and DST, whereas those assigned to the control group will be facilitated to do otherwise. Similar to the study by Lai et al [40], the sham protocol will include talking about overall issues related to wellness in later life (eg, diet and health, social activities, and family relationships) and creating a wellness scrapbook or journal.

**Figure 1:**
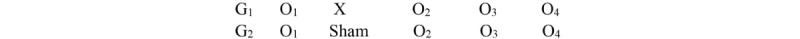
Data collection overview. G1: first group; G2: second group; O1: before the intervention; O2: middle of the intervention; O3: end of the intervention; O4: 3 months after the intervention; X: intervention.

As shown in Figure 1, data will be collected at baseline before the intervention, in the middle of the intervention, at the end of the intervention, and at 3 months after the intervention. The design of this study is depicted in scientific notation.

### Sampling and Recruitment Plan

Power analyses performed using G*Power 3.1.9 indicated that a total of 92 dyads (or 184 participants) are required to address our study objectives using a linear mixed model. This sample size estimate was based on a small effect size *(f*=0.22), a significance level of α=.05, β of .20, anticipated *r* over time of 0.30, and 5% attrition rate based on the findings of Chung [25], for 2-tailed tests.

Participants in this study will be community-dwelling older adults and undergraduate and graduate students (ie, young adult volunteers) enrolled at UTA. The eligibility criteria of the older adult participants include individuals who (1) are aged ≥65 years; (2) have cognitive impairment measured by the Ascertain Dementia 8 (AD8) [41]; (3) have decisional capacity as measured by the University of California, San Diego Brief Assessment of Capacity to Consent (UBACC) [42]; (4) are not participating in any other research protocol currently; and (5) can speak and understand English. The exclusion criteria for older adults are (1) participating in another trial, (2) having health issues that prevent them from participating in the study for 3 months, and (3) inability to commit to being available for the full 10 weeks of the intervention.

The older adult participants will be recruited through Tarrant County Meals On Wheels (MOW), which is a partner agency of the Area Agency on Aging of Tarrant County. Eligibility criteria to enroll in Tarrant County MOW is that a person must be homebound and have no one to help them on a regular basis. The Area Agency on Aging is very committed to expanding services and supports to people with ADRD, as many MOW clients with ADRD are living alone in Tarrant County. Moreover, studies suggest the need for additional research in the area of loneliness among clients using MOW [43]. Participant recruitment will begin with case managers and other MOW staff using their client database system to search for clients with ADRD who meet the study criteria. Next, case managers will contact potential study participants and then ask for permission to share their telephone number with a member of the UTA research team. The principal investigators (PIs) and graduate research assistants (GRAs) will follow-up with all the potential participants provided by MOW.

The young adult volunteers will include undergraduate or graduate students from UTA. The inclusion criteria for the young adult volunteers are the following: (1) aged 18 to 30 years, (2) currently enrolled as a student at UTA, and (3) can commit to be available for the full 10 weeks of the intervention. Young adult volunteers who have health issues or limited availability that prevent them from participating for the entire 10 weeks of the intervention will be excluded from the study. The study will be widely advertised across the UTA campus through avenues such as different student organizations, departmental social media accounts, departmental websites, and the offices of campus partners (eg, student health services, career services, campus recreation, and undergraduate honor program). Other recruitment efforts will include posting flyers across the campus, emailing student listserves, social media, advertising on television monitors located in buildings across campus, and classroom visits.

### Procedure

Before the intervention starts, the young adult volunteers in the intervention group and control group will be offered a mandatory separate group training through Microsoft Teams. Training for young adult volunteers in the intervention group will last 3 hours and be offered by LX, NLF, KMD, and BAT. Students will be provided with hard and electronic copies of the training materials. As part of the training, LX will cover an overview of the study and the intervention protocol, project timelines, responsibilities of the young adult volunteers, roles of the research personnel, and importance of human participants’ protection in the research activities. KMD will provide training related to ADRD, communication skills with older adults with ADRD, and how to deal with unexpected behavior or emotional symptoms of older adults during the intervention. NLF will cover how to communicate with older adults with ADRD. BAT will provide an overview of DST and details about DST techniques and demonstrate strategies for successful DST. Similarly, training for the young adult volunteers in the control group will be approximately 1 hour in duration and will cover human participants’ protection; study procedure (social visits); and explanation of the unstructured, nondigital record of the social visits. The trainings will be audio recorded and later analyzed for fidelity.

Subsequently, participants will participate in this telephone-based intervention. In this study, the young adult volunteers (in both the intervention and control groups) will talk with older adults through telephone calls each week for 10 weeks with the same process described in the following sections. Older adults in the intervention group will receive 10 sessions of life review with the trained young adult volunteers (1-1.5 hours each week for 10 weeks). During weeks 1 to 6, a life history interview will be conducted with different themes in each week: major turning points in life (W1), family history (W2), life or career accomplishments (W3), history of love and hate (W4), stress experiences (W5), and meaning and purpose of life (W6). Guidelines for conducting a life history interview will be prepared for the young adult volunteers based on the work of Watt and Cappeliez [44]. The implementation outline will encompass integrative and instrumental reminiscence techniques that aim to foster self-acceptance, conflict resolution, reconciliation, sense of purpose and self-value, recollection of effective coping strategies used in the past, and leveraging past experiences to address current challenges. After each session, the young adult volunteer will write a 1-page summary and reflect about the interview to help prepare for the subsequent DST in the later weeks. During weeks 7 to 10, the dyads will develop the DST together using tablets already owned by the UTA School of Social Work. The contents of sessions 7 to 10 will be structured as outline or script; plan and storyboard (W7); film and record (W8); evaluate, integrate, and finalize (W9); and publish or share (W10). It is acceptable if no filming or video aspect of the DST is conducted. Audio-based DST without video recording is still common, as it captures the voices that give life to the photos [27]. Photos obtained from the internet or the participants may be used as part of the DST to accompany the audio. For example, photos of the individual’s hometown, familiar places (ie, parks), familiar vacation locations (ie, monuments and historical markers), and so on, will help augment the voice recordings.

Older adults in the control group will have general social visit conversation with the young adult volunteers, and the content of weeks 1 to 6 will be based on general topics related to wellness in later life, instead of structured reminiscence guidelines. The topics of weekly social conversation are diet and health (W1), exercise or activity and health (W2), emotions and health (W3), religious or spiritual practices and health (W4), family or friend relationships and health (W5), and social activities or engagement and health (W6). For weeks 7 to 10, instead of DST, the young adult volunteer will work with the older adult to create an unstructured, nondigital record of the social visit related to wellness in later life (eg, wellness scrapbook or journal).

Every effort will be made to minimize risks by closely monitoring participants’ mood during weekly fidelity treatment checks. Weekly calls with the dyads in both the intervention and control groups will be conducted by the research team to closely monitor the participants’ mood and to serve as a fidelity check. These calls will include questions related to the study protocol (eg, topics covered, time spent, and challenges that occurred) and questions related to the well-being of participants (eg, How are you feeling? Do you need anything from the research team? and Are you experiencing any challenges?). If a study participant experiences emotional distress during the study, a clinical social worker and registered nurse practitioner, who are members of the research team, will offer consultation, and if necessary, they may refer study participants to additional services through the local Area Agency on Aging. Finally, individual retraining will be provided to the young adult volunteers in the case of problems with treatment fidelity.

To prevent contamination, the importance of confidentiality will be emphasized to participants in the intervention group at the beginning of the study. Participants (especially the young adult volunteers) in intervention group will be asked not to share the protocol with the participants in the control group for the duration of the study to minimize contamination. To further prevent contamination, the research team will check in with young adult participants in the intervention group 3 times (3 weeks, 6 weeks, and 9 weeks after the intervention starts) via telephone to ensure that no information has been shared with participants in the control group. Furthermore, young adult participants in the control group will also be asked 3 times during the study period whether they received any information related to the reminiscence and DST content, and this will be documented by the research team. In addition, substantial contamination can be tolerated within the usual individual randomized trial [45]. Finally, if contamination occurs, any documentation of hearing about or implementing reminiscence in the control group will be included as a covariate in the study analyses.

After the intervention, qualitative interviews with subsamples in the intervention group will be conducted. For the individuals in the highest 10% and lowest 10% of changes based on survey tests, we will conduct follow-up qualitative interviews to capture the details of their situations and present a more robust analysis, taking advantage of the strengths of both methods. The interview will last for 45 to 60 minutes and will be conducted by research teams through telephone calls.

### Measurement

#### Overview

The primary outcomes of the study will be the social and emotional well-being of older adults, and the secondary outcomes will be the benefits to the young adult volunteers. Social well-being will be measured by quality of life and loneliness, whereas emotional well-being will be measured by affects (positive and negative) and resilience. Regarding social well-being, quality of life is a relatively global and stable phenomenon that comprehensively measures people’s psychosocial well-being [46]. Recent studies suggest that loneliness, which represents the negative subjective experience of the inadequacy of social relationships, is a factor in evolutionary fitness across the life span [47] and is a good indicator of social well-being [48]. Regarding emotional well-being, PA and NA have been used as general dimensions to describe affective, emotional components of subjective well-being [49]. Resilience, as an important indicator of emotional well-being, assesses one’s ability to bounce back from or adapt to stressful situations or crises, such as having been diagnosed with ADRD [10,11]. Young adult volunteers will be asked about their attitude toward aging and knowledge and awareness about the ADRD disease. Demographic information about each dyad, such as age, sex, education, self-reported health, and so on, will also be collected in the pretest survey. A pilot test will be conducted on 3 volunteer dyads using assessment tools to evaluate participants’ possible overload impacts. In accordance with the input provided by pilot participants, the research team will review and make necessary modifications to the measurement selections within the initial 6 months of the project (preparation stage).

#### Primary Outcome Measurements for Older Adults

Quality of life of older adults in this study will be measured using Quality of Life–Alzheimer’s Disease (QoL-AD) [50]. The QoL-AD questionnaire consists of 13 items that cover various aspects including physical health, energy levels, mood, living situation, memory, family relationships, marital status, friendships, self-perception, ability to perform daily chores, engagement in enjoyable activities, financial situation, and overall life satisfaction. The higher the QoL-AD score, the better is the quality of life. Self-rating QoL-AD will be used, which had showed high reliability and validity [51,52].

Loneliness among older participants will be measured using the De Jong Gierveld Loneliness Scale [53]. This 6-item scale measures both emotional and social loneliness. This instrument has undergone validation in numerous countries, with validation studies encompassing various sexes and age groups and specifically including older adults. High scores indicate great loneliness. Reliability coefficients for both subscales were 0.81 to 0.85 [54].

The measurement of PA and NA in older adults will be conducted using the widely used Positive and Negative Affect Schedule (PANAS) [55]. PANAS assesses the respondent’s emotional state by inquiring about 10 positive and 10 negative emotions using a 5-point scale ranging from “Very slightly or not at all” to “Extremely.” This scale has demonstrated strong psychometric properties, with Cronbach α of .88 for PA and .87 for NA. Furthermore, PANAS has been validated across various cultures and languages worldwide, consistently exhibiting robust psychometric properties in multiple studies [49,56]. Total scores will be calculated for PA and NA, with high scores indicating more PA and NA, respectively.

The resilience of older adults will be measured using the Brief Resilience Scale [57]. The Brief Resilience Scale is recognized as one of the top and extensively endorsed resilience scales and recommended by Windle et al [58]. Extensive literature supports its high reliability and validity, as demonstrated by studies conducted by Smith et al [57,59]. A total of 6 items will be asked, and responses for each item ranges from 1 (*low resilience*) to 5 (*high resilience*). After reverse coding 3 items, total scores will be calculated for final analysis, with high score indicating more resilience.

#### Secondary Outcome Measurements for Young Adults

To measure the loneliness among young adult participants, we will use the De Jong Gierveld Loneliness Scale [53], following the same methodology used for measuring loneliness among older adults, as mentioned previously.

The Alzheimer’s Disease Knowledge Scale (ADKS) [60] will be used for the young adult volunteers. The ADKS is a comprehensive instrument consisting of a 30-item true or false scale. It can be completed in approximately 5 to 10 minutes and covers a wide range of domains, including risk factors, assessment and diagnosis, symptoms, disease progression, impact on daily life, caregiving, and treatment and management. Initial findings indicate that the ADKS exhibits satisfactory reliability, including test-retest and internal consistency and validity, including content, predictive, concurrent, and convergent validity. These results have been reported in studies conducted by Carpenter et al [60] and Spector et al [61]. Total scores will be calculated for final analysis, with high score indicating more knowledge.

To assess the young adult volunteers’ attitudes toward aging, the Fraboni Scale of Agism (FSA) will be used. The FSA, initially developed by Fraboni et al [62] and subsequently revised by Rupp et al [63], consists of 29 statements. Participants will rate their agreement on a 4-point Likert scale, ranging from 1 (*strongly disagree*) to 4 (*strongly agree*). The FSA has been widely used among college students and has demonstrated strong internal consistency and robust validity in previous studies [64,65]. Total scores will be calculated for final analysis, with high scores indicating more agism.

In addition, a 20-item questionnaire [25] will be developed to collect feedback from the young adult volunteers, covering (1) intervention preparation and organization, (2) intervention format and workload, and (3) perceived gains. Each item is rated on a 5-point scale (1=*strongly disagree* to 5=*strongly agree*), and the higher the score, the more positive the perception about the intervention.

#### Other Outcomes

Follow-up qualitative interviews will be conducted to explore participants’ overall experience with and perceptions about the intervention. On the basis of the results from the survey, we will select older and young adult participants who have the highest and lowest levels of changes in the outcomes for further qualitative interviews (10 dyads in total). Each generation’s experiences, perspectives, challenges, and suggestions regarding this intervention will be asked during these in-depth interviews.

### Data Collection Plan

Data will be collected at baseline, midpoint, end of the intervention, and 3 months after the intervention by telephone. A total of 3 GRAs will be trained to collect the data. Training on the assessment battery and interview techniques will be provided by PIs to GRAs. For the quantitative survey, after training, interrater reliability checks will be performed by 1 PI on pilot interviews of team members and participants (3 older adults and 3 college students not included in the final sample size), based on audiotaped interviews. For the qualitative interviews, training related to conducting interviews will be provided to GRAs, such as talking less and listening more, how to build rapport with the respondents, and how to handle unexpected emotions of participants. The GRAs will also pilot-test their qualitative interviewing skills based on role-plays with the research team. GRAs will have weekly supervision meeting with the PIs regarding participant recruitment and follow-up and survey assessments.

Several strategies will be used to track clients during the study and to minimize attrition. At the baseline and at each subsequent interview, participants will be asked to provide the names of 2 people who may know where they are if the researcher has difficulty in contacting them. We will also have the older participants’ MOW case managers as a backup point of contact. We will adopt the strategies of minimizing attrition in longitudinal studies [66], which results in 90% follow-up. These strategies (ie, getting several contacts, getting emails, reminder letters, and thank you letters) have proved to be useful in previous community-based studies conducted by the research team.

For individual missing responses on multiitem scales, we will compute scale scores by summing the available item scores as long as at least 80% of the item responses are available [67]. Missing data at the variable level will be handled through maximum likelihood imputation [67]. A concern with longitudinal research is that data are not missing randomly owing to systematic dropout. We will conduct attrition analyses to identify any factors systematically related to dropout and, if found, control for these effects in subsequent analyses. Any missing data that are random in nature, such as those that occur owing to random mistakes in data collection or entry and are not related to research staff or patient noncompliance, will be submitted to maximum likelihood estimation procedures to estimate those missing values. Maximum likelihood estimation for random missing data is a common and accepted procedure for missing data estimation [68,69]. If specific patterns of missing data are identified, we will further address missing values with multiple imputation. Multiple imputation will maximize accurate statistical inferences and allow for valid statistical inferences by appropriately accounting for the uncertainty caused by missing data [68,69].

### Statistical Analysis

After data are collected and entered into the statistical software, data quality will be carefully reviewed and then checked both automatically and manually. The review processes involved in data management encompass several steps. These include cross-checking the coding of observations or responses and identifying out-of-range values; ensuring data completeness; adding variable and value labels as needed; verifying random samples of digital data against the original data; implementing double entry of data; conducting statistical analyses such as determining the frequencies, means, and ranges to identify errors and unusual values; and rectifying any errors made during transcription. In addition, data will be thoroughly reviewed to address concerns related to the protection of human participants and confidentiality. This includes assessing the risk of potential disclosure of research participants’ identities, safeguarding sensitive information, and preserving the privacy of the collected data. As all the key concepts in the proposed study are measured using standard scales, reliability tests for each scale and test-retest reliability across the 4 surveys will be conducted.

For quantitative data analyses, descriptive analyses will first be conducted to provide the overall means and frequencies of key outcome variables for both older and young participants in the intervention and control groups. Fisher exact tests, Pearson chi-square tests, and independent samples *t* tests (2-tailed) will be computed to test for group differences that may be attributable to participants, such as age, sex, education level, income, and health conditions. If the groups significantly differ on any of these variables, the variable will be incorporated into the following analyses as a covariate, thereby controlling for potential influences that the covariate may have on the dependent variables. Moreover, in the event that at the culmination of this study, our sample sizes are unequal in the treatment groups or our outcome data are nonnormal, we will follow through with appropriate statistical corrections where necessary. Covariate-adjusted linear mixed models for repeated measures will be performed to compare the intervention and control groups over time for the major outcomes of older adults and young adult volunteers. The covariate will be any significant variable yielded from the univariate analyses, and the dependent variable will be the scores before the intervention, in the middle of the intervention, at the end of the intervention, and at 3-month follow-up. In this study, 95% CIs will be computed for each adjusted mean difference. The study α will be set to .05, with no adjustments for multiple comparisons owing to the preliminary nature of this study and the increased risk of type-2 error as a result of α adjustment [70].

Several different combinations of statistical results are possible. The results may indicate that the intervention and control groups significantly differ in outcomes among the older participants and the young adult volunteers. Another possibility is that differences may be discovered for one age group but not the others. Outcome measures may significantly change over time for none of the groups, some groups, or all groups. Another possibility is that the pattern of change over time for one group may not replicate in another group. This possibility, which would be identified via a significant interaction in linear mixed models, would require post hoc testing to investigate the nature of changes over time. In this case, groups will be isolated, and patterns of change will be identified via linear mixed models for repeated measures (with no between-participants factors). *For the qualitative data analysis*, the process will involve digitally recording and transcribing the qualitative interviews, followed by using conventional content analysis, as outlined by Hsieh and Shannon [71]. The research team, including LX and NLF, along with the research assistants, will independently code the data, forming the foundation for an initial coding framework. Subsequently, the researchers will convene to compare and establish consensus on the coding outcomes. Pertinent excerpts from the transcripts will be extracted and reorganized based on thematic relevance. The constant comparison process, as described by Glaser and Strauss [72], will be used to explore similarities and differences among participants who did and those who did not benefit from the intervention, generating subthemes within the broad topics. This comparative approach will enhance consistency and broaden the dimensions and comprehensiveness of each code. Eventually, the codes will be synthesized or combined into overarching recurrent themes. During the analysis stage, themes, research notes, and field notes will be reviewed using thematic charts. Multiple strategies, including peer debriefing, will be implemented to address potential investigator bias and enhance the study’s credibility. Maintaining comprehensive documentation will also ensure that an audit trail is available for future reference.

### Data Management and Participants’ Safety

The research team will be the sole individuals with knowledge about and access to the names of both the older and young participants. Although certain demographic information, such as age, sex, and racial and ethnic background, may be recorded, the questionnaires will not contain any participant identifiers such as date of birth or address. The preintervention and postintervention questionnaires will also be devoid of participant identifiers. To ensure confidentiality, participant names will be deidentified, with each individual assigned a unique study identifier or number. All data files will be securely stored in encrypted, password-protected folders on laptops provided by UTA. These laptops will also have encryption in accordance with the guidelines set by the UTA Office of Information Technology. Participant privacy will be diligently safeguarded, and records containing names and other identifiable information will be stored separately from the interviews, using password-protected, encrypted files on UTA-issued laptops. The PI (LX) and the UTA institutional review board (IRB) will bear the responsibility of ensuring participant safety and the attainment of the study’s scientific objectives. The research team possesses previous experience in organizing and managing clinical trials, including implementing data safety monitoring measures for interventions.

All the older adults and young adult volunteers will provide informed consent before participating in the study. Potential participants will be informed that refusal to consent to the study or withdrawing from the study will have no effect on the services that they currently receive or might receive through UTA (ie, young adult volunteers) or through MOW or the Area Agency on Aging (ie, older adults). Participants who provide informed consent will be contacted for baseline measurement and then randomized to 1 of the 2 treatment groups. The protocol and consent forms will be approved by the UTA IRB before conducting any study procedures. Student members of the research team will receive training regarding procedures required to obtain informed consent.

Human participants’ risk related to the study is minimal. The study carries potential risks related to the identification of depression or anxiety, as participants may become aware of their symptoms during the intervention sessions. This heightened awareness could lead to the recognition of distressing thoughts. In addition, there is a risk associated with the process of recalling past memories, as it may evoke difficult or emotional experiences for participants. Every effort will be made to minimize risks by closely monitoring the participants’ mood during weekly fidelity treatment checks. In the event that a study participant experiences emotional distress during the study, KMD, a registered nurse and gerontological nurse practitioner, along with NLF, a licensed clinical social worker, will be responsible for providing the necessary support and counseling to address the participant’s emotional well-being. They will also be available as needed for referral to the resources through the local Area Agency on Aging for the older adults and university services for the young adult volunteers, as needed.

### Ethics Approval

The study was approved by the UTA IRB on March 25, 2021 (2021-0206).

## Results

The study was funded in January 2021. Data collection started in May 2021 and ended in mid-2022. In total, 103 dyads were matched at the beginning of the intervention. Of the 103 dyads, 90 (87.4%) dyads completed the midtest survey and 64 (62.1%) dyads completed the posttest survey. Although a total of 92 dyads were planned to test the effectiveness of the intervention in this proposed study, the post hoc power analyses performed using G*Power 3.1.9 indicated that 64 dyads will yield sufficient power to test the interaction between groups over time (power=0.95), the changes over time (power=0.95), and the differences between the groups (power=0.71). These parameters are based on a small anticipated effect size (*f*=0.22), α=0.05, two-tailed, with measures correlated at r=0.30.

We also collected qualitative interview data for this study with selected dyads in the intervention group (8/27, 30%). Weekly reflection page from UTA student participants, weekly fidelity check forms, final digital story books, and final scrapbooks were also saved for future data analyses. We are currently engaged in data cleaning and analysis for both quantitative and qualitative data. One manuscript has already been published [73], and we anticipate publishing several more manuscripts reporting the results within the next 1-2 years.

## Discussion

### Principal Findings

The aim of this study is to develop and implement a 10-session intervention that combines reminiscence and DST techniques and foster connections between young adult college students and older adults with ADRD in a community setting. Through the collection of qualitative and quantitative data, this study seeks to evaluate the intervention’s potential efficacy in enhancing the social and emotional well-being of young and older adults and in improving the knowledge about ADRD and attitudes of young adults toward aging. By focusing on reminiscence and life review content, integrating DST technology, and including older adults with cognitive impairment, this study will contribute valuable and current data to address the existing research gaps.

Considering the multitude of psychological and mental health challenges experienced by older adults with ADRD, this study holds academic significance. By focusing on the social and emotional well-being of this population, the aim is to enhance their overall life satisfaction, positive emotions, and sense of purpose, factors that contribute to resilience and quick recovery from adversities linked to dementia diagnosis. Furthermore, this study adopts an innovative approach that combines the beneficial practice of reminiscence or life review facilitated by a DST product, alongside an intergenerational perspective, which also aims to address the social well-being (loneliness) issues of young adults. By engaging young adults as interventionists, this strategy empowers them to gain insights into older adults with ADRD, while reshaping their perspectives about aging. It also taps into their enthusiasm as volunteers, addressing staffing shortages and reducing expenses linked to professional interventionists. Consequently, it establishes a sustainable and economically viable intervention approach that is well suited for community-based environments. In addition, this study aims to address the limitations identified in previous studies, such as small sample sizes and the absence of a control condition.

### Limitations

A limitation of this study is its lack of representativeness. The older adult participants in this study only include MOW clients who generally have low income and are homebound. The young adult college students come from 1 public university in Dallas-Fort Worth area. Thus, the study has limited representativeness that cannot extend to other community-dwelling older adults and college students from other universities. Moreover, this study only focuses on the Dallas-Fort Worth area and thus, its results cannot be generalized to other cities, states, or regions. Future studies may consider scaling up this pilot into a trackable, replicable model in multiple sites to test the effectiveness of this intervention for older adults with ADRD. In addition, this protocol is proposed for in-person interactions between young and older generations. Owing to the risks and feasibility of conducting an in-person intervention during the COVID-19 pandemic, this project has to be tailored to telephone-based approach. Future study may consider comparing the effectiveness of interventions between in-person meeting versus telephone-based approach.

### Conclusions

Intergenerational reminiscence program that connects older adults with ADRD with young adult college student volunteers offers promising benefits for both younger and older generations in improving their social and emotional well-being. Future studies may consider scaling up this pilot into a trackable, replicable model that includes more participants with diverse background (eg, public vs private college and older adults from other agencies) to test the effectiveness of this intervention for the benefits of older adults with dementia and young adults.

## Appendix

Multimedia Appendix 1 Peer review comments from the Retirement Research Foundation for Aging.

## Abbreviations

ADKS: Alzheimer’s Disease Knowledge Scale
ADRD: Alzheimer’s disease and related dementia
DST: digital storytelling
FSA: Fraboni Scale of Agism
GRA: graduate research assistant
IRB: institutional review board
MOW: Meals On Wheels
NA: negative affect
PA: positive affect
PANAS: Positive and Negative Affect Schedule
PI: principal investigator
QoL-AD: Quality of Life–Alzheimer’s Disease
UTA: University of Texas at Arlington

## Data Availability

The data sets generated and analyzed during this study are available from the corresponding author upon reasonable request.
