# Supplementary material for: Reminiscence and Digital Storytelling to Improve the Social and Emotional Well-Being of Older Adults With Alzheimer’s Disease and Related Dementias: Protocol for a Mixed Methods Study Design and a Randomized Controlled Trial
Source: JMIR Res Protoc. 2023 Sep 7;12:e49752. doi: 10.2196/49752 (PMC10514775; doi:10.2196/49752)
Supplement: Multimedia Appendix 1 [file resprot_v12i1e49752_app1.pdf]

RRF Foundation for Aging

Below is text from an anonymous Peer-Review of a proposal titled, "Intergenerational Connections: Reminiscence and Digital Storytelling to Improve Social and Emotional Well-Being of Older Adults with Alzheimer's Disease and Related Dementias" that was submitted by Ling Xu, PhD. at the University of Texas at Arlington.

Potential impact and importance of proposal work and outcome

Due to the current absence of a curative or preventative treatment for dementia, there is significant need to develop psychosocial approaches that improve quality of life for a growing number of elders in our society—many of whom will struggle with the deleterious effects of social isolation. This project would test whether an intergenerational "intervention" (reminiscence and digital storytelling) delivered using college-aged volunteers can significantly improve a range of psychosocial factors relevant to quality of life (e.g. social wellbeing, loneliness, emotional wellbeing, etc.). It will also evaluate whether this intervention has reciprocal benefits for the young adult participants (e.g. knowledge of ADRD, attitudes towards aging, etc.).

While evaluation of psychosocial approaches in dementia care is intuitively important, there is presently a lack of strong data to support particular approaches. The main contribution of this study is its capacity to—as is recommended by the Cochrane Collaboration Review the authors cite on reminiscence approaches—bring a robust large-scale research design (RCT, mixed methods) to bear in establishing the benefits of a discrete intergenerational life story activity that is highly replicable. If carried out in full, this study will identify whether there are particular benefits to digital storytelling methods (or whether intergenerational interventions are more broadly therapeutic) and through what specific mechanisms the intervention may be therapeutic, e.g. improving loneliness, increasing resilience, etc. It will also evaluate reciprocal benefits for younger college-aged volunteers and use qualitative methods to explore underlying mechanisms of successful and unsuccessful dyads. All instruments proposed in this study are validated and reliable, and the strong methodology is suggestive that this study will be publishable in high-impact journals (I include some suggestions for the authors below).

I would assign this study high priority, as its rigor and the replicability of the intervention via national organizations like Meals on Wheels, Agencies on Aging, and undergraduate institutions are major strengths.

2. Relevance of proposed work to other work in field

Within the last several decades, there has been a rise in non-pharmacologic approaches in dementia care. These psychosocial activities (e.g. expressive arts, music, volunteering, etc.)—sometimes referred to as "socialceuticals"—have been shown to support QOL of persons living with dementia in ways that exceed current pharmacologic approaches and lack the expense and side effects of drugs. The investigators of this study are blending several approaches—intergenerational activities, reminiscence therapy, and digital storytelling—that have individually shown much promise in supporting QOL of persons living with dementia. In this way, the project does duplicate prior interventions but does so in a way that is novel by virtue of its syncretic approach and rigorous research design. While it could be argued that there is already ample support in the literature to recommend the activities pursued in this study, putting strong data behind common sense (i.e. "bringing the generations together around storytelling can be mutually beneficial") is an important contribution, and can be a necessary political tool in extending the reach of beneficial psychosocial approaches. Overall, the researchers are well-oriented to the larger aging research field and have designed a study that will make an important contribution.

3. Appropriateness and adequacy of the plan methodology

While the Covid-19 pandemic has disrupted intergenerational activities such as the one proposed in this study, the authors appear to have a strong contingency plan in place. That aside, the planned methodology is appropriate, thorough, and feasible. Their plan to closely monitor participant wellbeing through weekly fidelity treatment checks is especially admirable.

That said, it was not entirely clear how the researchers determined a 10-session intervention, and it will be important to make sure that this does not fatigue any of the participants living with ADRD. It was also not clear whether there would be a plan in place to help volunteers mitigate frustration that participants might experience related to their inability to recall facts from their past or otherwise express themselves coherently in the intervention or control activities. Relatedly, it may be wise to explicitly exclude participants who suffer from aphasias and may be less able to participate in intergenerational narrative or draw benefit from such a verbally-based activity.

With respect to replicability, the authors are pragmatic in partnering with the Agency on Aging and Meals on Wheels—two organizations with existing local/national infrastructure. Integrating college students to "deliver" the intervention is also favorable; however, it would have perhaps been fruitful to study whether high school-aged students could also deliver the study/control interventions to increase the replicability/scale of this work. While not every community will be anchored by a college/university, nearly all will have high schools (or organizations like Rotary, boy/girl scouts, 4H, etc.) that could be a

potential source of volunteers. Relatedly, Elizabeth Lokon—who has developed Opening Minds through Art—has recently bridged from using college undergrad volunteers for her intergenerational intervention to medical students and other students in health trainee programs. Methodologically, that might be a good future step to build towards, as there is a strong incentive for organizations training young healthcare workers within an aging society to integrate students into programming that can help enrich their understanding of people living with ADRD.

With respect to the publishing plan, because of the rigor of this study the authors ought to also consider high-impact geriatrics journals like *Journal of the American Geriatrics Society* and the *American Journal of Geriatric Psychiatry*—both of which have published studies on interventions like TimeSlips and Opening Minds through Art in recent years. Also, while it is a low-impact publication, they ought to consider the *Journal of Intergenerational Relationships*, as its audience is particularly receptive to innovations in intergenerational programming.

4. Strengths and weaknesses of proposal

<u>Strengths</u>

- The use of mixed methods and a robust RCT research design.
- Partnerships with Area Agency on Aging and Meals on Wheels.
- Qualitatively interviewing subsamples of participants who report the greatest and least changes in outcome measures.
- Addressing a research need identified by the Cochrane group.
- Potential replicability and reach of the intervention.
- Potentially contributing to growing evidence recommending the biopsychosocial benefits of non-pharamacologic interventions on the QOL for persons living with ADRD.
- Strong team with credentials and experience to carry out the study.

<u>Weaknesses</u>

- In the event Covid disrupts the intervention the contingency plans will allow the study to be carried out. However, one worries that the in-person, embodied nature of the narrative-based activity is the real source of biopsychosocial benefit. It was also not entirely clear whether elders would have sufficient tech support in the event a digital or phone-based form of correspondence is necessary.
- With respect to qualitative data, the researchers are using a fairly straightforward conventional content analysis that lacks the rigor of a grounded theory analysis. However, this is defensible in the context of a mixed methods study in which qualitative data will be used to help contextualize statistical findings and advance support model-building.
- As mentioned above, there is already a strong evidence-base to recommend the efficacy of reminiscence/intergenerational/storytelling approaches in dementia care. It can be fairly questioned whether more data are needed to substantiate common sense (i.e.

that bringing generations together to participate in a life-affirming activity would be mutually beneficial). However, this study does stand to contribute data that is particularly rigorous and meets an identified need in the field.

- At present, it is not entirely comprehensible how the national infrastructure of Meals on Wheels and the Agencies on Aging would be leveraged to disseminate/replicate the (hopefully successful) findings from this study.
- There are potential problems with reminiscence therapy. And while the authors cite Butler's continuity theory, more recent approaches have identified benefits for arts-based approaches (e.g. expressive art, imaginative storytelling) that do not rely so heavily on coherence and mnemonic performance, the failure at which can cause agitation, frustration, and sadness for persons living with dementia.

5. Budget

The budget seems reasonable, with most costs related to personnel. The request for project support (i.e. PhD student and 2 MSW grad students) seems warranted given the scale and ambition of this project, although one could objectively ask why more than one full-time student would be necessary. Material and supplies do not seem excessive and are justifiable.

6. Competency of the institution and staff

All of the study team members are qualified to undertake this study. All are experienced in the field (all will be at the associate professor level at the time of the study's initiation), and there is specific expertise in research design, statistical analysis, study recruitment and logistics, and delivery of the intervention(s). The request for project support (i.e. PhD student and 2 MSW grad students) seems warranted given the scale and ambition of this mixed methods project and its commitment to quality control (i.e. weekly fidelity treatment checks), although as mentioned above it could be asked whether more than one or two full-time support staff is necessary. This feels like the type of project that the team is poised to carry out well, and which could reasonably be developed into a successful NIH application.

7. Other observations/comments

As the authors argue, reminiscence approaches with persons living with ADRD do have a credible evidence base. However, one critique alluded to in the "Weaknesses" section above is that they can potentially cause frustration or agitation due to the inability to remember, and sadness at the evocation of emotionally-freighted memories. Other approaches—for instance TimeSlips, Opening Minds through Art, etc.—have evolved to focus more on spontaneous acts of expressive imagination and creativity rather than imposing the 'need to remember', and there is increasingly strong data substantiating those types of

approaches. That is not to say that this study’s intervention is ill-conceived; however, there would be value in potentially developing a study with multiple arms to test reminiscence against other less mnemonic approaches that are also intergenerational and narrative in nature (e.g. TimeSlips).

It would also be interesting—either in this study or a future study—to stratify student outcomes based on whether participants had a STEM or Humanities background in their training. Such a finding could help hone in on the “type” of student who (1) flourishes in this type of intergenerational activity or (2) grows the most as a result of their participation. Would likewise be beneficial to track long-term outcomes in students post-intervention (perhaps re-administering instruments 6 months or 1 year after the intervention ends?) and also descriptively tracking whether students pursue professional careers with aging persons or those living with ADRD.

It wasn’t clear whether the DST and/or scrapbook would be something that could be shared with family members of elder participants. While it seems that the DST product will be more carefully protected, if the scrapbook/journal is gifted to the resident it might be worth tracking what they do with them—for instance, do they share them with family members, caregivers, neighbors, etc.?

Loneliness is also increasingly—and tragically—pertinent to younger Americans. In 2017, 150,000 Americans died “deaths of despair” (alcoholism, suicide, drug overdose), and rising loneliness is increasingly viewed as playing a key role in the crisis. Recent CDC data from June 2020 showed that for Americans between 18-24 years old, 25.5 percent — just over 1 out of every 4 young Americans — said they had “seriously considered suicide in the past 30 days” (https://www.cdc.gov/mmwr/volumes/69/wr/mm6932a1.htm#suggestedcitation). Thus, it may be beneficial for future work (or this present study) to also evaluate loneliness for younger volunteers as a meaningful outcome, particularly in the Covid era.

One wonders whether the non-digital format (i.e. scrapbooking in the control group) may be more beneficial for a demographic that is less facile with technology. This finding would, in itself, be an important contribution.

It wasn’t clear if researchers would explicitly be collecting feedback from students on their training sessions, and it may be worth doing this, if only for program improvement.

8. Recommendation

I would strongly recommend that the RRF Foundation for Aging fund this work.

If you have any questions about this body of work or the review, please don’t hesitate to reach out to me.

Thank you,

Amy R. Eisenstein, PhD, FGSA
Senior Program Officer and Director of Research and Evaluation
Telephone: 773-714-8080
eisenstein@rrf.org
